# How Cumulative Statistics Can Mislead: The Temporal Dynamism of Sex Disparities in COVID-19 Mortality in New York State

**DOI:** 10.3390/ijerph192114066

**Published:** 2022-10-28

**Authors:** Ann Caroline Danielsen, Marion Boulicault, Annika Gompers, Tamara Rushovich, Katharine M. N. Lee, Sarah S. Richardson

**Affiliations:** 1 Harvard GenderSci Lab, Harvard University, Cambridge, MA 02138, USA; 2 College of Computing, Massachusetts Institute of Technology, Cambridge, MA 02139, USA; 3 Department of Linguistics and Philosophy, Massachusetts Institute of Technology, Cambridge, MA 02139, USA; 4 Department of Epidemiology, Rollins School of Public Health, Emory University, Atlanta, GA 30322, USA; 5 Department of Social and Behavioral Sciences, Harvard T.H. Chan School of Public Health, Boston, MA 02115, USA; 6 Department of Anthropology, Tulane University, New Orleans, LA 70118, USA; 7 Department of the History of Science, Harvard University, Cambridge, MA 02138, USA; 8 Committee on Degrees in Studies of Women, Gender, and Sexuality, Harvard University, Cambridge, MA 02138, USA

**Keywords:** COVID-19, gender, sex disparities

## Abstract

Overall, men have died from COVID-19 at slightly higher rates than women. But cumulative estimates of mortality by sex may be misleading. We analyze New York State COVID-19 mortality by sex between March 2020 and August 2021, demonstrating that 72.7% of the total difference in the number of COVID-19 deaths between women and men was accrued in the first seven weeks of the pandemic. Thus, while the initial surge in COVID-19 mortality was characterized by stark sex disparities, this article shows that disparities were greatly attenuated in subsequent phases of the pandemic. Investigating changes over time could help illuminate how contextual factors contributed to the development of apparent sex disparities in COVID-19 outcomes.

## 1. Introduction

It is well established that COVID-19 outcomes, like many health outcomes, differ across demographic groups: COVID-19 incidence and mortality vary by socially relevant factors such as age, comorbidity status, occupation, socioeconomic status and race/ethnicity [1,2,3,4,5,6,7,8,9,10]. Researchers have observed that outcomes also differ by sex, with initial assessments claiming that men were dying at twice the rates of women [11]—a claim frequently cited to support the hypothesis that biological sex-related variables are the primary explanation for men’s greater susceptibility to severe COVID-19 [12,13,14,15,16,17]. However, if this striking disparity in fact reflects just a temporal slice of the pandemic, such conclusions could be misleading. Here, we investigate variation in sex disparities over time and, therefore, across shifting social, political and economic circumstances, using the case example of New York state (NYS), a global index site in the pandemic [18].

In this analysis, we examine how the number of male COVID-19 deaths in excess of female deaths accrued dynamically over time between March 2020 and August 2021 in NYS. While, overall, more men than women died from COVID-19 during the period of observation, attending to how mortality patterns vary across time uncovers patterns that are otherwise obscured, potentially revealing how socio-contextual factors may contribute to sex disparities in infectious disease outbreaks [19]. Rigorous application of these methods in future analyses of infectious disease pandemics can improve apprehension of how time-varying social, economic and political factors like stay at home orders, mask mandates, and school closures interact with biological variables as well as with gender differences in occupation, health behaviors, and pre-existing conditions to produce gender/sex differences in risk of COVID-19 exposure and death.

No studies have examined the distribution of sex disparities in COVID-19 mortality over time in NYS. In August 2021, NYS was in the top quintile for cumulative sex disparity in COVID-19 mortality in the U.S. [20]. Despite accounting for 6% of the total U.S. population, as of August 2021, the state contributed 8% of the country’s COVID-19 fatalities and 9% of the total U.S. difference between male and female deaths [21,22]. Below, we describe large changes in mortality by sex across different phases of the pandemic in NYS. Specifically, we find that the majority of the excess male deaths accrued in the seven weeks following the first recorded COVID-19 death in the state. Furthermore, we show that the difference between the COVID-19 mortality rate for men and women ranged from 4.61 to 310.92 per 100,000 person-years across different segments of the eighteen-month study period. Appreciation of this temporal dynamism in sex disparities in COVID-19 mortality in this specific locality can help illuminate the role of social and contextual factors–rather than biological sex alone–in producing sex disparities in health outcomes.

## 2. Materials and Methods

We obtained sex-disaggregated COVID-19 mortality data for NYS from the US Gender/Sex COVID-19 Data Tracker (hereafter referred to simply as the Tracker) for the seventy-five weeks spanning from 14 March 2020 through 28 August 2021 [20]. The Tracker was developed by the Harvard GenderSci Lab and recorded weekly COVID-19 case and fatality data disaggregated by sex. The Tracker’s fatality data include all individuals categorized as women or men who died from COVID-19 in NYS, as reported by the New York State Department of Health [23]. The methodology of the Tracker and how it was developed has been described elsewhere [19,20]. Deaths with sex categorized as “unknown” or “other” constituted 0.03% of the total and were excluded from the analysis. Data used in this analysis were publicly available, de-identified, and are exempt from IRB oversight. The first available weekly data point (27 April 2020) included all COVID-19 deaths that occurred in NYS up to that point in time (the very first death was recorded on 14 March 2020 [24]).

For each week in the period of observation, we subtracted the count of women’s deaths that had occurred until that point in time from the corresponding count of men’s deaths. The weekly men-to-women differences were plotted over time, expressed as a percentage of the cumulative difference between male and female deaths recorded by the end of the observation period. We then inspected the graph visually and identified two time periods (14 March 2020 to 4 May 2020 and 8 December 2020 to 9 May 2021) in which the difference in mortality by sex increased, and two time periods (5 May 2020 to 7 December 2020 and 10 May 2021 to 28 August 2021) in which it appeared to remain stable. To further characterize the variation in the COVID-19 sex disparity over time, we computed mortality rates by sex and mortality rate differences for each of the four time periods identified. We used the 2015–2019 5-Year American Community Survey population estimates for NYS as population denominators [25]. Mortality rates by sex are reported per 100,000 person-years. We divided the total number of deaths in each sex stratum during a given time period by the corresponding number of person-years, which we computed by multiplying the sex-specific population by the duration of the time period [5]. Mortality rate differences were computed using women as the reference population.

## 3. Results

A total of 43,522 individuals are recorded as having lost their lives to COVID-19 between 14 March 2020 and 28 August 2021 in NYS and were included in the analysis. Of these, 19,227 (44.2%) are women and 24,295 (55.8%) are men (Table 1), a difference of 5068 deaths between the two groups. The cumulative mortality rate over the entire period of observation was 131.07 (95% CI: 129.22–132.29) per 100,000 person-years for women and 175.56 (173.35–177.77) for men, corresponding to a mortality rate difference of 44.49 (41.61–47.37).

**Figure 1:**
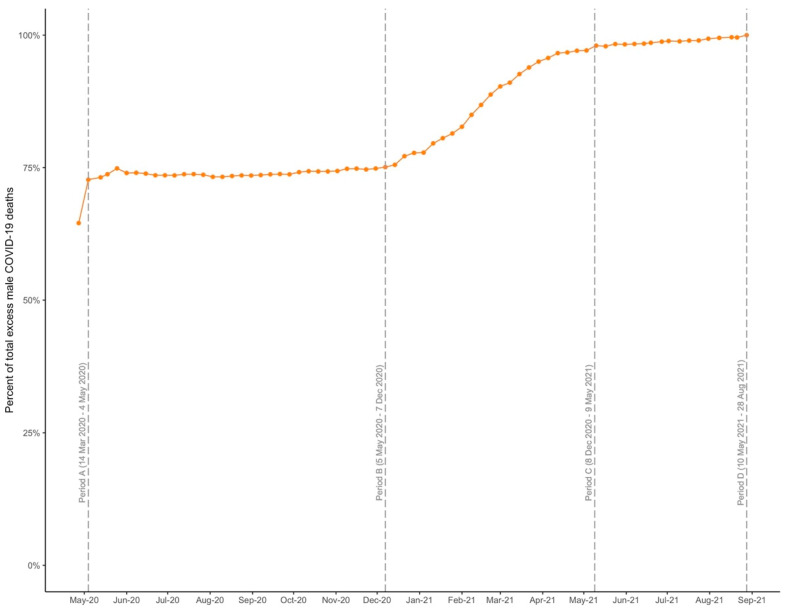
Cumulative excess male COVID-19 deaths by week as a proportion of total excess male COVID-19 deaths during the study period (14 March 2020–28 August 2021).

Our analysis reveals that sex disparities varied dramatically across the pandemic. Each weekly data point in Figure 1 represents the percentage of the cumulative difference between men and women’s deaths that was accumulated to that point in time. For example, on the graph, 9 May 2021 corresponds to the data point 98.0%. This indicates that on 9 May 2021, 98.0% of the 5068 total excess male deaths recorded on the last day of observation had taken place.

The most notable observation to emerge from Figure 1 is that by 4 May 2020, 72.7% (*n* = 3686) of the cumulative difference between men and women’s deaths had already been accumulated (Period A in Figure 1). This figure should be contextualized alongside the fact that 44.1% (*n* = 19,180) of deaths recorded over the entire period of observation occurred during the same time period. In Period A, the mortality rates for both men and women were higher than in subsequent time periods: 550.89 (538.63–563.16) per 100,000 person-years among women and 861.81 (846.01–877.61) among men. The corresponding mortality rate difference by sex was 310.92 (290.91–330.92).

The flattening of the curve in Figure 1 after 4 May 2020 signals an abrupt decrease in sex disparities in COVID-19 mortality. Between 5 May 2020 and 7 December 2020 (Period B in Figure 1), the number of deaths was 4040 among men and 3920 among women, a difference of 120 between the two groups. Therefore, despite accounting for 18.3% of total deaths, Period B contributed to only 2.4% of the 5068 male excess deaths recorded by the end of the observation period. Between 5 May 2020 and 7 December 2020, the gap between the mortality rate for women (65.82 [95% CI: 63.76–67.88]) and men (71.90 [69.69–74.12) decreased drastically compared to Period A, resulting in a mortality rate difference of 6.09 (3.06–9.11).

The second time period that sizably contributed to the cumulative difference in deaths by sex occurred from 8 December 2020 to 9 May 2021 (Period C in Figure 1). In period C, a total of 15,156 (34.8%) deaths took place, with the difference between male and female fatalities being 1162: 22.9% of the total difference on the last day of observation. The mortality rate was 166.94 (163.03–170.86) for women and 206.35 (201.88–210.83) for men, a difference of 39.41 (33.46–45.36) deaths per 100,000 person-years between the two groups.

Between 10 May and 28 August 2021 (Period D in Figure 1), we observe a decrease in COVID-19 mortality. Mortality rates were at their lowest for both women (18.56 [17.03–20.10]) and men (23.17 [21.41–24.93]), with 100 more deaths being registered among men compared to women (2.0% of the total difference). During Period D, the mortality rate difference by sex was 4.61 (2.27–6.95).

## 4. Discussion

Using unique sex-disaggregated longitudinal data from the US Gender/Sex COVID-19 Data Tracker, our analysis shows significant variation in the magnitude of sex disparities in COVID-19 mortality in NYS over time. That is, sex disparities in COVID-19 mortality did not remain stable over time, nor were they accumulated in a linear manner. Rather, shorter time periods during which sex disparities sharply increased (e.g., period A and C in Figure 1) were followed by longer periods in which the sex disparity remained close to parity (e.g., period B and D).

While the cumulative mortality rate difference over the entire period of observation was 44.49 (95% CI: 41.61–47.37) deaths per 100,000 person-years, it stayed consistently below this figure over the majority of the study period (6.09 [3.06–9.11] in Period B, 39.41 [33.46–45.36] in Period C and 4.61 [2.27–6.95] in Period D). The sex disparity was greatest early in the pandemic between 14 March and 4 May 2020, when the mortality among men was 310.92 (290.91–330.92) deaths per 100,000 person-years higher than the mortality among women. During this period, 72.7% of the cumulative difference between male and female deaths was accrued, creating a gap in mortality rates that continues to affect the cumulative sex disparity to the present day. Examining sex disparity data only cumulatively obscures the fact that a sizable proportion of the sex disparity was accumulated during a discrete period of time. This case study of NYS demonstrates that attending to variations over time is essential to testing the sensitivity of apparent sex differences to time-bound contextual factors.

Many gender-related social and demographic factors could produce such variation in sex parity [9,26,27,28,29]. Notably, as of September 2021, New York City (NYC) accounted for 80.7% of COVID-19 male excess deaths in NYS, while only accounting for 53.1% of all deaths in the state [22]. As is well known, NYS and in particular NYC were hit by COVID-19 especially early and severely compared to the rest of the US. During this time, NYS instituted several provisions to curb the spread of the pandemic. For example, non-essential businesses were closed on 22 March, individuals were banned from gathering on 24 March, and face masks were required in public places beginning 17 April [30,31]. The precipitous decline in male mortality observed after 4 May 2020 follows the progressive implementation of pandemic-control provisions, thereby supporting existing research suggesting that gendered behavioral, occupational, and structural factors play a central role in determining disparities in COVID-19 mortality [9,19,26].

The relative increase in male mortality during Period C coincided with the second major surge of COVID-19 in NYS, as well as with the large-scale roll-out of COVID-19 vaccines. Gender/sex patterns of vaccination linked to age, demographics, occupation, health behaviors, and other social variables could have affected COVID-19 disparities during this period. For example, women accounted for the majority of vaccine recipients in NYS in the early phases of vaccine rollout [32], which could have resulted in protection against COVID-19 death varying by sex. Moreover, the gradual lift of pandemic-control provisions in NYS starting in February 2021, including the reopening of public school and the extension of opening hours of gyms, bars and restaurants [33,34], could have replicated some of the conditions that contributed to the stark sex disparities in Period A.

Sex-disaggregated COVID-19 mortality data in NYS were not reported in conjunction with any other demographic variables, but data on sex as it interacts with other factors such as age, race/ethnicity, socioeconomic status, occupation, and comorbidity are crucial to better understanding sex disparities in COVID-19 [9]. For example, Rushovich et al. showed that aggregate sex comparisons without intersectional analysis of race/ethnicity obscured very high COVID-19 fatality rates for Black women compared to both white women and white men, and relatively low fatality rates for white men, compared to Black men [26]. A limitation of the present case study is that data from the US Gender/Sex COVID-19 Data Tracker are not available stratified by age. Therefore, age-adjusted rates could not be computed, despite age being a critical factor in vulnerability to COVID-19 [1,7]. Nonetheless, crude mortality data provide an indication of how sex disparities unfolded over time and can prompt questions related to the gendered socio-contextual factors that might have contributed to them.

## 5. Conclusions

Overall, our findings demonstrate that in New York State, an early and severely-impacted global index site in the pandemic, sex disparities in COVID-19 mortality have not remained stable across time and were greatly attenuated after the initial, most acute and deadly phase, prior to the introduction of public health controls. This suggests that sex disparities in COVID-19 mortality may be context-dependent and socially mediated to a significant extent, and may be ameliorable by public health policies. The social patterning of health outcomes, including for COVID-19 sex disparities, has been widely documented. Without dismissing a possible role for biological variables, our findings underscore the importance of investigating contextual factors in relation to changes in the magnitude of sex disparities for understanding and addressing their root causes [29,35].

More broadly, the case of the temporal dynamism of sex disparities in COVID-19 mortality in New York State provides a remarkable example of the perils of crude sex comparisons that are insensitive to patterns of variation. In fact, nearly three-quarters of the total sex disparity in COVID-19 mortality in this index locality accumulated in the first seven weeks of the pandemic, with sex disparities never returning to this magnitude in the sixteen months following. As we argue here, cumulative statistics obscure this variation and may generally misguide the apprehension of COVID-19 sex disparities. We hope that future research will replicate similar analyses across other global index sites (e.g., the Lombardy region in Italy and the city of Wuhan in China) and investigate how socio-contextual factors in those localities may have affected the development of sex disparities over time.

## Figures and Tables

**Table 1:** COVID-19 mortality counts, rates and rate differences in New York State by time period, disaggregated by sex.

Time Period	Count (%)				Rate (95% CI) *		Rate Difference **
	Women	Men	Total	Difference	Women	Men	
Period A: 14 March 2020– 4 May 2020	7747 (40.4)	11,433 (59.6)	19,180 (44.1)	3686 (72.7)	550.89 (538.63, 563.16)	861.81 (846.01, 877.61)	310.92 (290.91, 330.92)
Period B: 5 May 2020– 7 December 2020	3920 (49.2)	4040 (50.8)	7960 (18.3)	120 (2.4)	65.82 (63.76, 67.88)	71.90 (69.69, 74.12)	6.09 (3.06, 9.11)
Period C: 8 December 2020– 9 May 2021	6997 (46.2)	8159 (53.8)	15,156 (34.8)	1162 (22.9)	166.94 (163.03, 170.86)	206.35 (201.88, 210.83)	39.41 (33.46, 45.36)
Period D: 10 May 2021– 28 August 2021	563 (45.9)	663 (54.1)	1226 (2.8)	100 (2.0)	18.56 (17.03, 20.10)	23.17 (21.41, 24.93)	4.61 (2.27, 6.95)
Entire observation period: 14 March 2020– 28 August 2021	19,227 (44.2)	24,295 (55.8)	43,522 (100.0)	5068 (100.0)	131.07 (129.22, 132.29)	175.56 (173.35, 177.77)	44.49 (41.61, 47.37)

* Rate per 100,000 person-years. ** Rate differences are calculated with women as the reference population.

## Data Availability

Data available in a publicly accessible repository that does not issue DOIs. Publicly available datasets were analyzed in this study. This data can be found here: https://www.genderscilab.org/gender-and-sex-in-covid19 (accessed on 20 August 2022).
